# Towards a Fishing Pressure Prediction System for a Western Pacific EEZ

**DOI:** 10.1038/s41598-018-36915-x

**Published:** 2019-01-24

**Authors:** Megan A. Cimino, Mark Anderson, Travis Schramek, Sophia Merrifield, Eric J. Terrill

**Affiliations:** 1 Scripps Institution of Oceanography, University of California San Diego, La Jolla, 92093 CA USA; 2 Institute of Marine Science, University of California Santa Cruz, Santa Cruz, 95064 CA USA; 3 Jacobs School of Engineering, University of California San Diego, La Jolla, 92093 CA USA

## Abstract

Fisheries management faces numerous monitoring and enforcement challenges that are becoming more complex as fish stocks are depleted; and illegal, unregulated, and unreported fishing becomes more sophisticated. For remote island nations, the challenges are compounded by a loosely understood association of pelagic stocks to the ocean environment, and the tyranny of distance in monitoring and surveilling large exclusive economic zones (EEZ). An approach to ocean conservation is establishing protected areas, with the Pacific island nation of Palau as a leader with the recently established National Marine Sanctuary, which closes 80% of their EEZ to commercial fishing in 2020. Here we present an EEZ-wide analysis of Palau commercial fishing over a 6-year period (2011–2016), and develop a system for predicting fishing activity accounting for oceanic variables, climate indices, and vessel flag. Linking pelagic habitat to fishing activity provides high-resolution decision aids for management, highlighting the need for EEZ-specific analyses in addressing fisheries.

## Introduction

There is an unprecedented need for forecasts to guide fisheries management and monitoring, control and surveillance (MCS) as overfishing and illegal, unreported, and unregulated (IUU) fishing has become an increased problem with cascading biological, economic, and security effects^1–6^. It is often challenging to find and deter these unlawful activities because of the vastness of the ocean and the resources required for open ocean surveillance are both expensive and limited in availability. Recent studies of global fishing patterns reveal fishing hotspots in relation to fishing methods, the environment, and cultural effects, such as ocean temperature and holidays^7–9^. However, it is unclear if generalized global or regional fishing patterns accurately reflect the specific fishing footprint in each country’s exclusive economic zone (EEZ) that may be dependent on country-specific regulations, trade-relationships with foreign countries, and geo-specific ocean habitat that varies in time. To combat IUU fishing and effectively manage the oceans, which is EEZ-dependent due to the host-nation’s laws, EEZ-specific fishing patterns need to be assessed to create useful forecasting tools that can enable precision management^10–12^.

**Figure 1:**
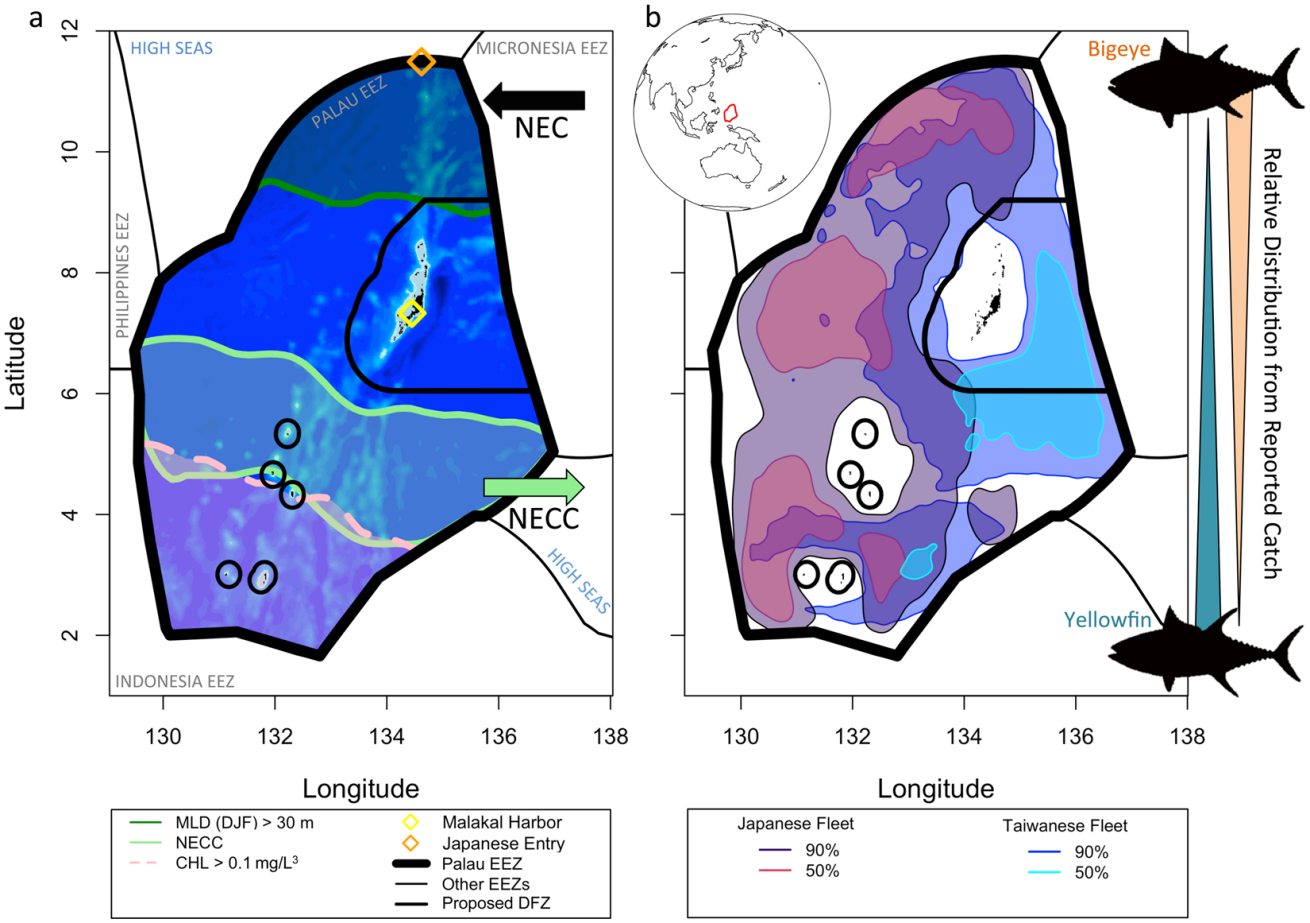
In the tropical western Pacific, the Palau Exclusive Economic Zone borders Indonesia, Philippines, Federated States of Micronesia and two high seas pockets, and has distinct biophysical characteristics that influence fishing patterns. (**a**) The mean region with the eastward flowing North Equatorial Counter Current (NECC, u velocity >0.1 m/s), the region influenced by the westward North Equatorial Current (NEC), high Chlorophyll-a concentrations (CHL) and deep winter (December, January, February - DJF) mixed layer depths (MLD) are shown. Bathymetry is shown in the background. (**b**) Regions fished by the Taiwanese and Japanese tuna longline fleets, with catch being mostly bigeye in the north and yellowfin in the south. The kernel density estimate for the 90% and 50% contour represents the range and core of fishing locations.

The world’s largest stocks of tuna occur in Pacific Island waters and over half of the tuna caught in the Western Pacific are from small island nations^13,14^. However, enforcement capabilities within these countries are often resource-limited and undersized relative to the enormous ocean jurisdictions resulting from political boundaries between distributed island chains; thus, introducing a vulnerability to IUU activities^15^. Having a deep cultural heritage for ocean conservation, these countries are strong advocates of a ‘Blue Economy’ and the sustainable use of ocean resources for economic growth, and are turning towards an increased reliance on green tourism dollars to recapture lost income from commercial fishing licenses^16,17^. Palau has emerged as a global leader in ocean conservation (receiving the 2012 Future Policy Award for developing the world’s best policies to protect oceans and coasts) and is the focus of our study. In 2009, Palau created the world’s first Shark Sanctuary that banned all commercial shark fishing and in 2015, Palau passed into law a National Marine Sanctuary that designates 80% of their EEZ as a sanctuary no fishing zone, with the remaining 20% to serve as a domestic fishing zone (DFZ) beginning in 2020 (Fig. 1a). The motivation behind the sanctuary is a cultural desire to protect their resources, which are also vital for the tourism industry that supports 56% of Palau’s gross domestic product^18^, compared to fishing that contributes approximately 3%^19,20^. For the sanctuary to be effective, it must be enforced^21–23^, which emphasizes the need for integrated MCS for their maritime EEZ of >600,000 sq. km. To understand the temporal and spatial fishing trends around Palau, we combined Vessel Monitoring System data (VMS, a commercial fisheries surveillance system operated by the Forum Fishing Agency, and mandated on all commercial fishing vessels) with concurrent ocean state information to identify fishing patterns and their dependence on the environment.

## Results

### Fishing and environmental patterns in the Palau EEZ

We processed six years of VMS data, containing time records of 1.2 million discrete vessel locations, and developed algorithms to separate fishing activity from transiting behaviors. We focused on areas with fishing activity or “effort” (ex. Fig. S1), and identified the flag states present and fishing methods used. Vessels from seven different flag states fished in Palau’s EEZ from 2011 to 2016 with 99.73% of the fishing locations from longline fleets (100 vessels total) and only 0.26% from purse seiners (11 vessels, Table S1). Fishing was dominated by two flag states, the locally-based foreign longline fleet operated by Taiwan, and the offshore-based longline and purse seine fleet from Japan (Fig. S2). Our analysis goal was to determine the relationship between these two dominant fleets and the ocean environment with a high degree of statistical confidence.

Combining fishing locations from all 6 years revealed spatial patterns by flag state, gear type and species caught. For indeterminate reasons (but hypothesized to relate to fuel savings), the Taiwanese and Japanese longline fleets partitioned the Palau EEZ longitudinally with Taiwanese vessels fishing mainly in the east and Japanese vessels in the west (Fig. 1b). The three core fishing regions for Japanese longliners were distributed evenly north to south while the core fishing region for Taiwanese longliners was to the southeast of Palau, near their homeport in Malakal Harbor (Fig. 1a,b). Both fleets caught more bigeye tuna (*Thynnus obesus*) in the north and more yellowfin tuna (*T. albacares*) in the south^24^ (Fig. 1b). The Japanese purse seine fleet (6 vessels, Table S1) fished the southeast corner of the Palau EEZ, most frequently from March to July, creating a spatial segregation in areas of highest purse seine and longline fishing effort (Figs S3, 1b). Purse seiners caught mostly skipjack tuna (*Katsuwonus pelamis*)^24^. The Japanese purse seine fleet was a small proportion of all fishing locations (0.099%, Table S1), and during the 20 months when a vessel was present in the EEZ, each fished for approximately 4.1 ± 3.0 days (Fig. S2). Therefore, purse seine vessels were excluded from further analyses to focus on drivers of longline efforts.

**Figure 2:**
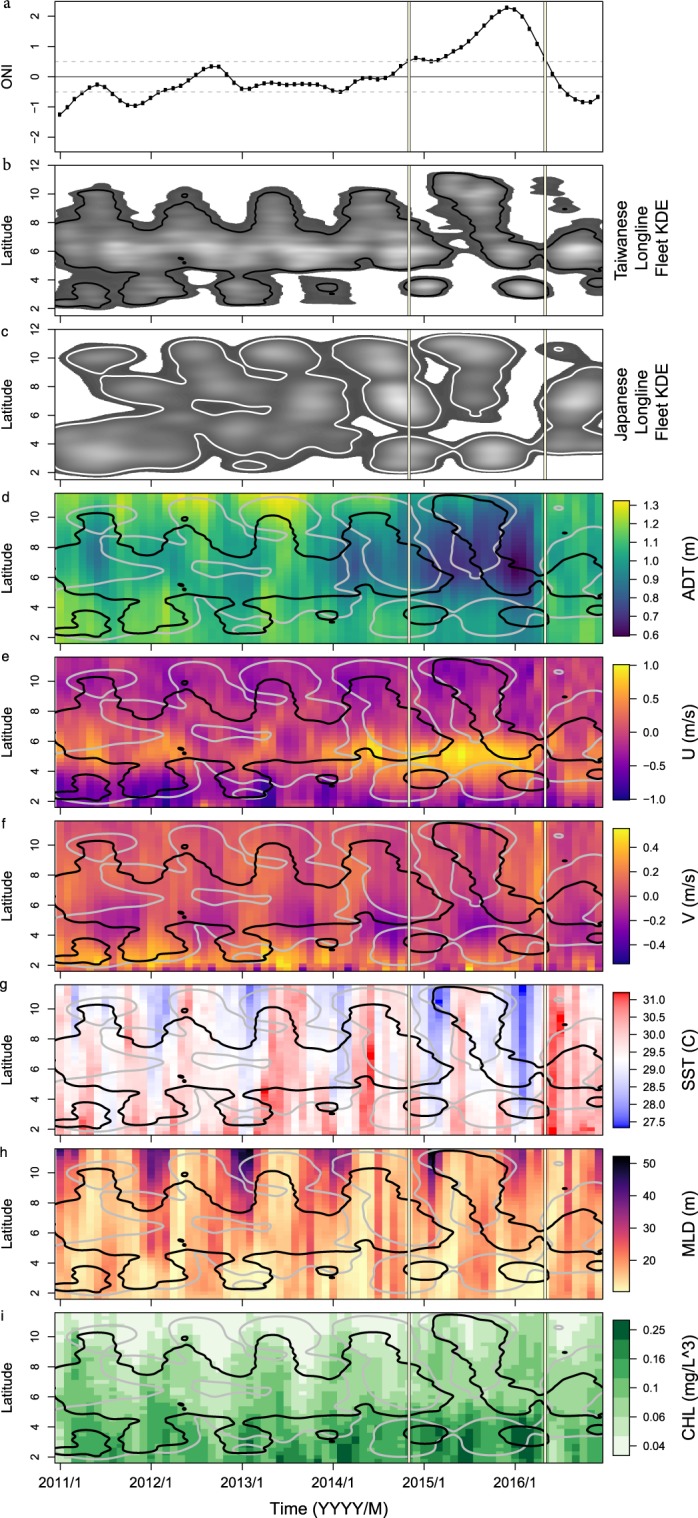
Hovmoller diagrams showing seasonal patterns in fishing locations and oceanography in the Palau Exclusive Economic Zone. (**a**) The Oceanic Niño Index (ONI), where values > 0.5 are El Niño events (vertical bars) and values < −0.5 are La Nina events. (**b**) The kernel density estimate (KDE) of Taiwanese and (**c**) Japanese longline fishing vessel locations. (**d**) Absolute dynamic topography (ADT), (**e**) U velocity, (**f**) V velocity, (**g**) sea surface temperature (SST), (**h**) mixed layer depth (MLD), and (**i**) Chlorophyll-a concentrations (CHL). The 90% KDE of Taiwanese and Japanese fishing locations (from **b**,**c**) is overlaid as black and gray contours, respectively, in (**c**–**i**).

While Taiwanese and Japanese fishing locations spanned the entire EEZ from 2011 to 2016 (Fig. 1b), fleet efforts were observed to shift south to north by season, winter to summer, respectively. Both flag states fished the mid to southern portion of the EEZ from October to March and fished the mid to northern portion of the EEZ from April to September (Fig. 2bc). This pattern was consistent from 2011 to 2014 but was disrupted during the El Niño period (Nov 2014-May 2016, Oceanic Niño Index (ONI) > 0.5, Fig. 2a), where fishing extended further north during mid-2015 and further south at the end of 2015 and into 2016. The rapid response of fishing locations to El Niño conditions suggests a tight correlation exists between the environment, the fish, and the fishing industry.

A summary schematic using data during a continuous El Niño neutral period (−0.5 < ONI < 0.5, March 2012-Oct 2014, Fig. 2a) revealed major ocean features within the Palau EEZ that influenced fishing locations (Fig. 1a). The North Equatorial Counter Current (NECC) is a strong eastward flowing current typically located south of Palau that changes orientation and strength throughout the year^25^ (Fig. 2e). Core fishing regions do not overlap with the center of the NECC (Fig. 1a,b). Chlorophyll-a concentrations (CHL) were generally of low amplitude year round but highest (>0.1 mg/L^3^) in the southern portion of the EEZ, often south of the core of the NECC. During winter months (December, January, February - DJF), there was a cyclical deepening of the mixed layer depth (MLD) from ~10 to >30 m in the northern portion of the EEZ.

Physical and biological ocean properties had apparent seasonal cycles and latitudinal gradients, and were altered during the El Niño period (Fig. 2). There were evident seasonal cycles during winter where sea surface temperature (SST) was colder (Fig. 2g), MLDs were deeper (Fig. 2h) and CHL was lower (Fig. 2i). Latitudinally, SST was colder in the north without any sharp spatial gradients within the domain as might be seen in higher latitude waters where SST fronts provide signatures of advected water masses and solar forcing of the remotely sensed ‘skin layer’ is less dominant. MLDs were deeper in the north, and CHL was higher in the south. Longitudinal (U) current velocities were strongest at ~5°N (Fig. 2e), revealing the NECC. There were concurrent changes in water properties during the El Niño period, including lower Absolute Dynamic Topography (ADT) (Fig. 2d), increased eastward (+U) velocities, decreased SST, deeper MLDs and higher CHL (similar to the 1997-98 El Niño^26^).

To characterize suitable fishing habitat, we built four separate boosted regression tree statistical models^27–29^ relating satellite observations of the ocean environment to the presence/absence of vessels and number of fishing days (hereafter, count) for Taiwanese and Japanese longline fleets. Presence/absence models included information on whether a vessel was present or absent within each grid cell while count models provide information on fishing effort per grid cell via the number of fishing days across all vessels in that area each month. Our models performed well according to our diagnostic measures (high area under the receiver operating characteristic curve (AUC), low root mean squared error (RMSE), Table S2). Count models performed better than presence/absence, explaining 47% and 22% of the deviance for Taiwan and Japan (Table S2). These performance metrics are comparable to studies using similar approaches to study animal distributions^30–33^, which along with human behavior can be challenging to predict. The Taiwanese models had more explanatory power, likely due to a larger sample size of fishing observations and consistent fishing pressure over time compared to the time variable Japanese fleet (Tables S1, S2). Both presence/absence and count models for Japanese and Taiwanese vessels reliably reproduced spatial patterns and seasonal variability in fishing locations (Movies 1–4, Figs S4, S5), where mean fishing probability and number of fishing days (Fig. S5) were significantly higher inside the 90% contour of observed fishing locations compared to outside the contour (t-test, p < 0.05). These validation results are an essential first step towards science-based ecosystem monitoring and management of an EEZ-specific fishery.

The variables that were important in driving fishing patterns were fairly similar for presence/absence and count models for each flag state. For the Taiwanese models, the most important predictor variable was distance to Malakal Harbor, contributing ~40%, while the remaining predictors (U/V velocity, MLD, ADT, and CHL) contributed 10–15% to the models (Figs S6, S7). For the Japanese models, the main predictors were ADT and MLD, contributing roughly 20% each, while the remaining variables (Distance, U/V velocity, and CHL) contributed 12–17% to the models (Figs S6, S7).

Predictor variables of the physical environmental appear relevant to both the fish and the fisher. The Taiwanese fleet generally fished in waters that were ~100–500 km from their homeport in Palau (Figs S6, S7), perhaps related to shorter travel time (reportedly 5 hrs from port^34^) and fuel savings. In comparison, distance from where the Japanese fleet likely entered the EEZ did not contribute as strongly to the models, perhaps because Japanese vessels routinely stay at sea for long periods having already traveled at least 2500 km from their homeports in Japan. Japanese vessels fished both near the entry point as well as >700 km away. Both flag states avoided fishing in areas with high eastward velocities (the NECC), indicating this is either a challenging fishing environment or unlikely place to catch fish. Higher ADT (mainly 0.8–1.2 m) was preferred for both the Japanese and Taiwanese fleet, as low values were a proxy for the disruptive El Niño conditions. High ADT is directly related to sea surface height, which is an indicator of the depth of the thermocline in the vicinity of Palau^35^. Notably, SST was not an important predictor, albeit SST and SST fronts are commonly a proxy for tuna habitat due to thermal tolerances and the tendency of fish to aggregate along temperature gradients^36–39^. For example, adult bigeye, yellowfin, and skipjack tuna have mean temperature tolerances between 9–26 °C, 16–27 °C, and 16–28 °C, respectively^19,40^. While there were weak meridional gradients in SST over the range of Palau’s EEZ (Fig. 2g), the strong meridional gradients in the temperature structure of the ocean interior (as represented by MLD and ADT in Figs 2d,h) explain the difference in predictive capabilities of these physical variables. Catch data suggests tuna species could be partitioning their habitat by temperature^40^ with bigeye, yellowfin and skipjack often caught in the north (cooler), mid to southern, and southern (warmer) portions of the EEZ, respectively^24^. Therefore, time-dependent temperature may be a better indicator of species caught than fishing location.

**Figure 3:**
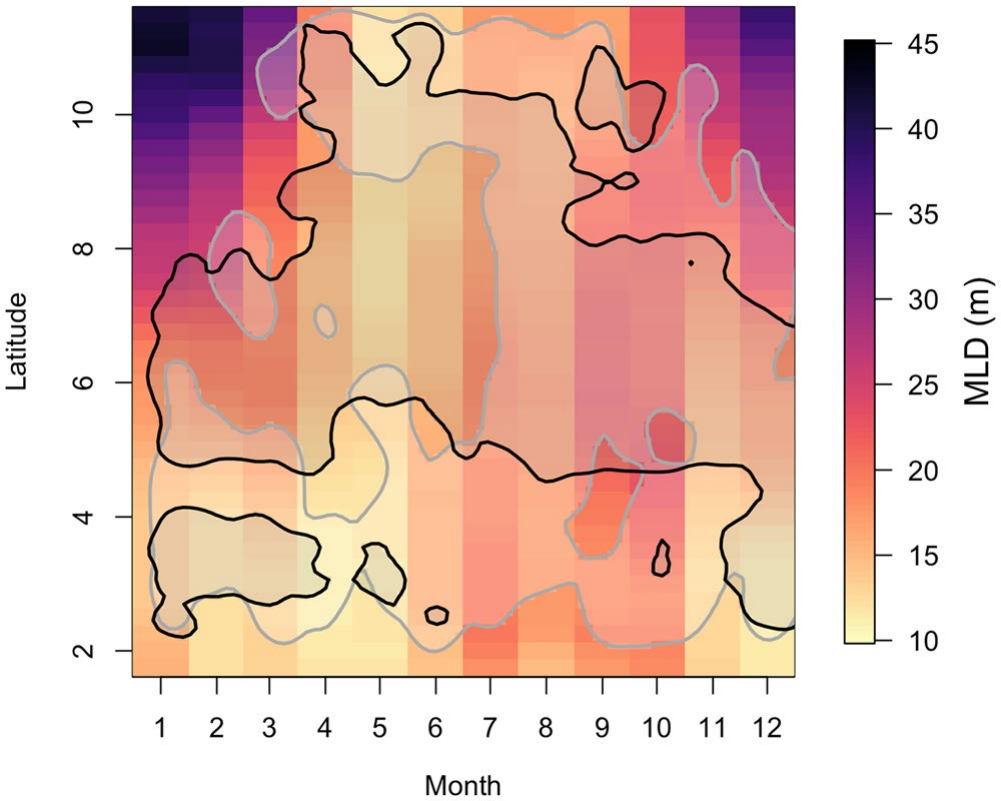
Hovmoller diagram showing monthly average mixed layer depth (MLD) across the latitudes spanning the Palau Exclusive Economic Zone. The 90% kernel density estimate contours for Taiwanese and Japanese fishing locations are in black and gray. MLD and fishing locations span the neutral phase of ENSO.

Predictor variables of the biological environmental may be indicative of trophic linkages or fish food sources. Vessels were observed to fish in waters with generally low CHL, a proxy for primary production and the base of the food web. CHL is usually indicative of higher trophic levels^36,37^ but this surprising negative relationship can be explained by the north-to-south gradient in CHL (Fig. 1a) and the north-to-south pattern in fishing effort (Fig. 2a,b). Vessels fished in the northern EEZ in summer when CHL peaked in the southern EEZ, and vessels fished the southern EEZ in winter when CHL was seasonally lower (Fig. 2i). Additionally, fishing occurred more frequently in regions with shallow MLDs (<30 m), which were deepest at northern latitudes in winter months (Figs 1a,2h, 3). Vessels avoided fishing in regions with deep MLDs year-round (Figs 2h, 3). MLD along with ADT may be related to the depth of the thermocline or deep scattering layer (often 250–500 m during the day^41^ that contains tuna prey). The daily vertical movements of larger tuna (especially bigeye) are known to mirror the depth of the deep scattering layer while smaller skipjack and yellowfin tuna cannot swim in the cold waters below the thermocline^19,42^. A shallower thermocline may confine young tuna to shallower waters while a shallower deep scattering layers could be indicative of shorter vertical migrations and tuna being more aggregated, resulting in increased catch rates^43^. As many EEZs have no physical boundaries compared to ocean basins, our results emphasize that while specific ocean properties, such as SST and CHL, may be common regional or basin scale drivers of tuna habitat, the drivers may be substantially different at the local EEZ scale. Furthermore, a global study showed little relationship between fishing vessel behavior and environmental drivers^9^ whereas fishing in the Palau EEZ was strongly tied to seasonal ocean features, suggesting that if intricacies within each EEZ are ignored, then key patterns may be overlooked.

**Figure 4:**
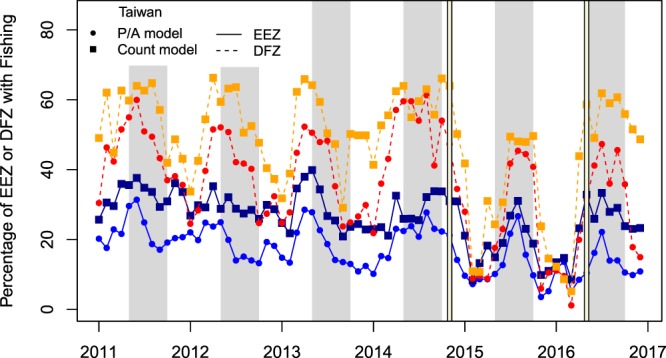
The percentage of the Exclusive Economic Zone (EEZ) and proposed Domestic Fishing Zone (DFZ) with suitable fishing conditions predicted by the presence/absence (P/A) and count model for Taiwan. Suitable conditions have fishing probabilities >0.5 (P/A model) or ≥1 vessel fishing (count model). For clarity the four groupings are colored. The percentage of the EEZ with suitable fishing conditions from the count model is in dark blue, the percentage of the EEZ with suitable fishing from the P/A model is in royal blue, the percentage of the DFZ with suitable fishing from the count model is in orange, and the percentage of the DFZ with suitable fishing from the P/A model is in red. The vertical yellow lines represent the El Niño period and the gray bars highlight May to October when fishing conditions often peak.

Models for Taiwanese vessels (the dominant fleet) revealed temporal patterns in the percentage of suitable fishing habitat in the EEZ and proposed domestic fishing zone (DFZ, Palau’s sanctuary containing 80% of the EEZ) (Fig. 4). Seasonally, the predicted highest percent of suitable fishing habitat was in the summer (often May to October) when vessels were predominately fishing in the northern portion of the EEZ. In general, 30–60% of the DFZ (~30,000–60,000 km^2^ of 99,739 km^2^) was predicted to have suitable fishing habitat while 10–30% of the EEZ (~60,000–180,000 km^2^ of 608,927 km^2^) was predicted to be suitable each month. During the El Niño period, these percentages dropped to below 10% in winter for both regions, and to ~25% and 45% of the EEZ and DFZ in summer, with an overall average 40% drop in suitable habitat. The count and presence/absence models were highly correlated within and between the EEZ and DFZ (Pearson’s *r* > 0.80, p < 0.05), suggesting these predictions are robust. Similarly, an average of 50 vessels fished in Palauan waters each month from 2011 to 2014 (Fig. S2). This number decreased to 30 vessels during the El Niño period, an overall 40% decrease in fishing effort, and increased after El Niño conditions dissipated (Fig. S1). There was a significant positive correlation between the number of vessels in the EEZ and the percentage of the EEZ with suitable fishing habitat, according to both presence/absence and count models (*r* = 0.52 & *r* = 0.54, p < 0.05), suggesting more vessels fished when there was more suitable habitat to catch fish.

The El Niño-Southern Oscillation (ENSO) is known to alter the distribution, abundance and catch rates of tuna throughout the Pacific^9,43,44^. Around Palau, El Niño conditions likely decreased tuna abundance/availability, as indicated by the decreased fishing effort^7^ (Fig. S1), predicted fishing habitat suitability (Fig. 4) and reported catch in 2015^20,24,34^. Therefore, El Niño conditions could have serious food security and economic consequences for Palau and export countries, especially when fishing is limited to the DFZ. Global studies often ignore relevant climate indices and environmental factors^9,45^ that drive fish distributions and are necessary for providing useful and actionable information to managers. These infrequent events should be planned for and can be monitored using a fishery’s forecasting approach based on real-time oceanographic observations and forecasting systems.

## Conclusion

Our results reveal the spatial, temporal and interannual variation in fishing efforts in Palau’s EEZ in relation to oceanography, flag state of the fishing fleet, and climate events. Oceanographic conditions were a predominant predictor of the seasonal variation in fishing effort after separating the data by their flag states, which is often not considered in larger-scale/global analyses. Fishers appear to be highly tuned to the environment, as fishing pressure was observed to respond to El Niño with little lag, revealing they rapidly respond to variation in conditions to seek out profitable fishing environments.

Our models provide the groundwork for a quantifiable fore/nowcast system, which can guide MCS to combat the multifaceted IUU fishing problem, and provide important insights to fisheries management. An important next step would be to further substantiate our models with available catch and landing information. Currently, the location of VMS-tracked vessels can be used as a guide to search for IUU fishing, but when Palau’s sanctuary prohibits fishing throughout most of the EEZ, there will be nothing to direct surveillance efforts. This shift emphasizes the need to understand environmental linkages of fishing patterns for guiding or queuing future MCS when commercial fishing ends. In addition, Palau is more vulnerable to IUU activities being located adjacent to two high seas pockets, allowing vessels to engage in unlawful activities with quick egress out of controlled EEZs. Therefore, this forecasting system can be used for queuing patrol vessels, satellite imagery, and/or airborne surveillance to regions with the highest probability of fishing, significantly improving their efficiency since in general only ~30% of the EEZ has suitable fishing habitat at any time. Forecasting tools can also inform fishers of the most probable areas to catch fish, allowing them to optimize their efforts. As managers look ahead, the approach outlined for fishing habitat predictions can be extended to estimate landings and participants utilizing the DFZ, and consider how to best expend resources as fishing habitat varies. The migratory nature of tuna both seasonally and interannually also suggests cooperation between neighboring EEZs, and even across ocean basins, is necessary because poor management in one EEZ can affect stocks in another. While it is unrealistic to expect all island nations in the Western Pacific to immediately create no-take protected areas, their cooperation is essential in curtailing IUU activities. The analysis framework presented also provides a methodology for island nations to develop both the protected area and DFZs, as a high-resolution examination of past fishing patterns will support the design of DFZs in areas with suitable fishing habitat year round to ensure local economic and food security.

## Methods

### Data Sources and Gridding

We obtained environmental data within the Palau EEZ that corresponded to the time period of our VMS records. We selected our suite of biological and physical parameters based on quality, availability and relevance to fish or fishers in the tropical Pacific.

The Oceanic Niño Index (ONI) was obtained from the NOAA Climate Prediction Center (http://origin.cpc.ncep.noaa.gov/products/analysis_monitoring/ensostuff/ONI_v5.php). The ONI is a measure of the El Niño-Southern Oscillation, where values > 0.5 are indicative of El Niño events and values < −0.5 are La Niña events. The ONI provides monthly reports, consisting of three month running averages.

Surface velocities (u and v current speed and direction) were obtained from Ocean Surface Current Analysis Real-time (OSCAR), which combines sea surface height, surface vector wind and sea surface temperature from various satellites and *in situ* instruments (https://www.esr.org/research/oscar/). Data are reported on a 1/3 × 1/3 degree grid with a 5-day resolution. Monthly averages were computed.

Monthly sea surface temperature (SST) and chlorophyll-a concentrations (CHL) at 4 km resolution were obtained from the MODIS Aqua satellite (http://oceandata.sci.gsfc.nasa.gov/MODISA/Mapped/Monthly/4km/chlor/, http://oceandata.sci.gsfc.nasa.gov/MODISA/Mapped/Monthly/4km/SST/). All statistical analyses using CHL data were computed in log_10_ units.

Monthly Absolute Dynamic Topography (ADT) and mixed layer thickness defined by density or mixed layer depth (MLD) were downloaded from the E.U. Copernicus Marine Service Information (http://marine.copernicus.eu/). ADT is the sea surface height above the geoid and was obtained from the Global Ocean Gridded SSALTO multimission ground segment/Data Unification and Altimeter Combination System (DUACS) (L4, delayed time, 0.25 × 0.25 degree grid) (http://misgw-sltac.vlandata.cls.fr:45080/thredds/dodsC/dataset-duacs-rep-global-merged-allsat-phy-l4-v3). MLD was on a 0.083 degree × 0.083 degree grid and was obtained from the Global Ocean Physics Analysis and Forecast (http://opendap-glo.mercator-ocean.fr:8080/thredds/dodsC/global-analysis-forecast-phy-001-024-monthly).

Because these environmental parameters were measured on different spatial scales, each parameter was interpolated to the same 0.125 × 0.125 degree grid and data outside of the Palau EEZ was removed. This grid resolution was useful because it was small enough that it did not obscure ocean features that may be a cue for fish or fishers and it was large enough that it allowed us to pool VMS observations for modeling purposes (discussed below).

Bathymetry was obtained from ETOPO1, which is a 1 arc-minute global relief model (https://www.ngdc.noaa.gov/mgg/global/global.html). The proposed National Marine Sanctuary locations were obtained from the Palau National Marine Sanctuary Office (https://www.protectedplanet.net/palau-national-marine-sanctuary-marine-sanctuary).

We also tested if fishing vessels fished in waters closest to their homeport. For the Taiwanese fleet, distance was calculated from each grid cell in the Palau EEZ to their homeport in Malakal Harbor, Palau. The Japanese fleet is based in Japan; therefore, we calculated distances from each grid cell to a north entrance point (Fig. 1a), an estimated entry point of Japanese vessels into Palauan waters.

### Identifying Fishing Activity from Vessel Data

Understanding patterns of the fishing behavior of individual vessels, as well as entire fleets, has been facilitated by the Automatic Identification Systems (AIS, a mandatory collision avoidance system for sea-going vessels with publically available data) and the Vessel Monitoring System. VMS is the preferred method for studying vessel tracks because AIS data density over the open ocean depends upon satellite coverage and vessels may deliberately cheat by turning off AIS transponders or falsifying data^46^. The Palau VMS database contains the geographic position, date, time, identification number and vessel name for vessels within the Palau EEZ. The government of Palau has identified the authors to be Maritime Domain Awareness Partners, providing permission for the access of VMS data from the Pacific Island Forum Fisheries Agency from 2011 to 2016. The fishing gear used, flag state, and other vessel information was obtained by linking VMS data to vessel registries using the vessel identification number (https://www.ffa.int/node/42/). Our statistical analyses are restricted to the fishing vessel locations in aggregate in order to protect the identities and fishing patterns of individual vessels.

Our goal was to identify vessel behaviors (transiting vs. fishing) to focus on locations where vessels were likely catching fish (similar to^47–51^). We attempted to use a number of machine learning approaches and data filtering techniques to identify fishing patterns. These approaches were marginally successful, partly due to not having catch or observer data to train the model. Processing the data was also complicated by the fact that vessel report rates ranged from 30 minutes to 4 hours, with more frequent reporting in 2014–2016 compared to 2011–2013. It is nearly impossible to identify patterns in tracks with only 6 vessel locations per day. Therefore, we opted to using a simplified approach that allowed data from all years to be utilized without having to omit sparse observations, mainly from 2011–2013. Our approach removed all transiting behaviors and assumed the remaining tracks represent fishing behavior, or that the vessel was loitering in the vicinity of fishing habitat. We refer to these as fishing locations with the caveat being we have no data to confirm if fish were actually caught.

For each vessel, our fishing detection algorithm cycled through a number of steps using Scilab 6.0.0 software for numerical computation^52^. Vessels with sample sizes less than 100 locations were excluded from the analysis, due to challenges in discerning behavior from so few observations. First, in our algorithm, improbable locations were removed from the dataset, where reported speed was greater than 12 kts. Then, the speed and heading was estimated along the vessel’s track. Second, we found lines of constant speed and heading. We considered long transits to be characterized by tracks greater than 8 hrs at a constant course within 30 degrees and a constant speed within 2 kts. These long transits were removed from the dataset. Third, because fishing is prohibited within a 50 nm radius of Malakal Harbor (Republic of Palau Public Law No. 6–36 amended Chapter 1 of Title 27) and vessels behaved irregularly near Palau (the Taiwanese port being in Malakal Harbor, Palau), all data within a box surrounding Palau was removed (6.6–8**°**N, 135–133.8**°**E). The remaining data were grouped into tracklets with a new tracklet being created if there was a gap of more than 6 hours between points. The tracklets were upsampled to 30 minutes to put all data on the same time scale.

From these assumed fishing locations, we determined the number of vessels by gear type (longline and purse seine) and flag state, and the percentage of fishing locations by country and gear type. Because Japanese and Taiwanese vessels overwhelming dominated (98.7%), our analyses focused on these two flag states.

The VMS data was then matched to our environmental grid to create two datasets on a monthly scale, (1) vessel presence or absence and (2) the number of fishing days summed across all vessels within each grid cell (referred to as ‘count’ below). This cataloging was done separately for the Taiwanese and Japanese fleet given the potential of their homeport to influence fishing locations. Finally, the presence/absence and count datasets were matched to the corresponding environmental data from each grid cell for each month from 2011–2016. This approach is similar to past studies, where AIS or VMS information are combined with concurrent environmental observations to identify areas frequently utilized and key environmental features, such as current fronts^53^, chlorophyll fronts^54^ and SST preferences^55^.

### Modeling Fishing Habitat - Boosted Regression Trees

We used a multi-model approach to predict whether fishing was present or absent and the amount of fishing effort via the number of vessels that fished each month. This approach provided robust results on the environmental parameters that were important to producing preferable fishing habitat. We ran four different models: (1) presence/absence of Taiwanese vessels, (2) presence/absence of Japanese vessels, (3) number of fishing days summed (count) across all Taiwanese vessels, and (4) count across all Japanese vessels. Our approach is very similar to species distribution or niche modeling techniques where tagged tuna or vessel catch locations were matched to environmental features to predict species habitat preferences (ex.^36–39^).

We used a popular modeling approach, called boosted regression trees (BRTs), where many simple models are iteratively fit to a random subset of the data (bag fraction) and are combined to better estimate a response^27,28^. BRTs are a machine learning, predictive modeling technique that is considered to have greater predictive performance and ability to elucidate complicated relationships among many variables when compared to other traditional approaches (such as, generalized linear or additive models)^27,29,33,56^. BRTs can deal with missing data, outliers, non-linearity, multicollinearity, irrelevant predictors, and violations of traditional statistical assumptions including independence of data and unequal variances^29,32^. BRTs combine many models (each classification tree) and include stochasticity to reduce model variance and improve predictive performance. BRTs are a common approach to elucidate ecological relationships, and have been previously used to look at fishing effort^31^, fisheries data^30,32^ and to create dynamic habitat management tools^57^ similar to our study.

We followed established protocols for fitting BRTs^29,32^ and used the brt.functions package in R, produced by^29^ (R Development Core Team 2017). A Bernoulli (binomial) distribution was used for presence/absence models and a Poisson distribution for count models. We used a tree complexity of 3 (number of nodes controlling which interactions are fitted) as two and three-way interactions among variables may be important but higher-orders are likely unnecessary (as in other studies^30,31^). A bag fraction (percentage of data used for model building in each step) of 0.7 was used^30^; it has been suggested that using 50–75% of data in building each tree gives the best results^29^. The learning rate (contribution of successive trees to the growing model) or shrinkage for each model was tuned so that no less than 1,000 trees (or iterations) were included in the final model (following methods outlined in^29^).

All model results were validated using a set of diagnostic metrics and visualized. Because our dataset was large ( >200,000 locations from 2011–2016) and predictive modeling can be computationally expensive, we randomly divided our dataset into fourths and then BRTs were run on each of the four partitions. We validated all models on an internal and external dataset using 25% and 75% of the data, respectively. External validation was used to determine the predictive power of the resulting model on previously unseen data^58^. Internal validation was done using 10-fold cross validation (to avoid overfitting^59^) on 25% of the dataset and external validation evaluated the performance of the model on the left out 75% of the data. For the four data partitions, each final model (containing 25% of the data) was projected onto all of the data. After all four partitions were run, we mapped and calculated the mean and standard deviation in model predictions for each month for a visual comparison with the actual data (see Movies 1–4). We also created Hovmöller diagrams that grouped mean and standard deviation in predictions by month and latitude (by 0.25 degree resolution) to compare with known fishing locations. To interpret our internal models, we quantified the relative influence of predictor variables and constructed partial dependency plots to show the influence of a variable after accounting for the average effects of all other variables in the model^29,59^. These partial plots may not represent the comprehensive effects of each variable on the response, especially if strong interactions or correlations between predictor variables are present. For presence/absence models, model accuracy was determined using the area under the receiver operating curve (AUC) measurement. A model with perfect performance would have an AUC of 1, whereas a model performing no better than random would have an AUC of 0.5^60^. For count models, the root mean squared error (RMSE) expressed as a percentage of the maximum was reported to compare predictions to observations^30^. For both model types, the proportion of deviance explained was reported^61^. These three measures of performance (AUC, RMSE and explained deviance) are common methodology^62^. From the internal and external validation, we reported the mean and standard deviation of AUC or RMSE and deviance explained.

All predictor variables were used in initial model building. SST was dropped in all final models, as it was identified as an unimportant variable from recursive feature elimination, a process analogous to backward selection in regression^29^. BRTs generally ignore non-informative predictors but, given our large dataset and many predictor variables, it was useful to eliminate an unimportant term to avoid overfitting. Multicollinearity can lead to unstable parameter estimates and inflated standard errors when correlation coefficients are greater than 0.7^63^. Pairwise Pearson correlation coefficients for all predictor variables in our models were <0.4; therefore, all selected variables contributed unique information to the final models.

We further evaluated the performance and utility of the Taiwanese model predictions by calculating the percentage of the EEZ with suitable fishing habitat and compared this value to the numbers of vessels that fished in the EEZ each month. Suitable habitat for the presence/absence model was determined by the number of grid cells where fishing probability was >0.5 for each month. For the count model, we used the number of grid cells where ≥1 vessel was predicted to be fishing each month. We then determined the percentage of grid cells that met these thresholds. Furthermore, with the proposed closure of the EEZ and creation of a domestic fishing zone (DFZ), it would be useful to know the proportion of suitable fishing habitat within the proposed DFZ each month. Therefore, we used the Taiwanese models to calculate the percentage of the DFZ with suitable conditions each month. Vessels fishing in the proposed DFZ will have a home port in Malakal Harbor; thus, the Taiwanese model that uses distance from Malakal Harbor as a predictor variable was deemed appropriate. In addition, because the Japanese fleet was a small (11.89%) proportion of all fishing locations in the Palau EEZ (Table S1) and because our model predicted a comparatively small proportion of the EEZ to be suitable for the Japanese fleet (Supplement movies), we decided to utilize the Taiwanese model for this analysis.

### Kernel Densities Estimates

We compared the spatial fishing locations of Japanese and Taiwanese vessels using a two-dimensional kernel density estimation (KDE) method with an axis-aligned bivariate normal kernel^64,65^. We show the 90% and 50% contours to represent the main and core fishing locations for both countries, commonly utilized thresholds in animal ecology^66,67^. Similarly, we computed the 90% kernel density estimate for Japanese and Taiwanese fishing locations by time, from 2011–2016, and latitude to compare with biological and physical variables over the same temporal and spatial scales. This same contour was used to visually and statistically validate our BRT model predictions for both presence/absence and count models by aggregating the predictions by month and latitude in the Hovmöller diagram. We determined if BRT model predictions inside the 90% contour were significantly different from predictions outside of the contour for each of the four models using a 2-group Welsh t-test. Finally, the 90% KDE contour was computed for fishing locations by month and latitude, including only data during the neutral phase of El Niño, to compare with MLDs over the same time and space scale. These analyses were performed in R (R Development Core Team 2017).

## Supplementary information

Movie 1

Movie 2

Movie 3

Movie 4

Supplemental Information

## Data Availability

The environmental datasets used in this study are publicly available online as stated in the methods. The VMS dataset analyzed in this study is not publicly available to protect the vessel’s identity and the information can be commercially sensitive. The authors’ use of the raw VMS data was under an agreement between the Government of Palau and Scripps (E.J.T.).
