# Supplementary material for: Towards a Fishing Pressure Prediction System for a Western Pacific EEZ
Source: Sci Rep. 2019 Jan 24;9:461. doi: 10.1038/s41598-018-36915-x (PMC6345951; doi:10.1038/s41598-018-36915-x)
Supplement: Supplementary file 5 — Supplemental Information [file 41598_2018_36915_MOESM5_ESM.pdf]

Towards a Fishing Pressure Prediction System for a Western Pacific EEZ

Megan A. Cimino, Mark Anderson, Travis Schramek, Sophia Merrifield, Eric Terrill

SUPPLEMENTAL INFORMATION

Supplemental Table 1. The number of longline and purse seine fishing vessels and the percentage of fishing locations from each flag state from 2011 to 2016 in the Palau EEZ.

Flag State	Number of Longline (Purse Seine) Vessels	Percentage of Longline (Purse Seine) Fishing Locations
China	1 (0)	0.15 (0)
Papua New Guinea	0 (1)	0 (0.0057)
Belize	2 (0)	0.080 (0)
Vanuatu	2 (0)	0.90 (0)
Philippines	0 (4)	0 (0.16)
Japan	35 (6)	11.89 (.099)
Taiwan	60 (0)	86.72 (0)
**Total**	**100 (11)**	**99.73 (0.26)**

Supplemental Table 2. Boosted regression tree model performance diagnostics for monthly presence/absence and count models (the number of fishing days summed across all vessels) for Taiwanese and Japanese vessels.

	**Internal Validation**		**External Validation**	
**Model**	**AUC**	**Deviance explained (%)**	**AUC**	**Deviance explained (%)**
**Taiwanese Presence/Absence**	84.25 ± 0.14	27.56 ± 0.38	84.51 ± 0.07	28.15 ± 0.14
**Japanese Presence/Absence**	78.19 ± 0.36	14.19 ± 0.45	78.55 ± 0.33	14.78 ± 0.45
	**RMSE (% of max)**	**Deviance explained (%)**	**RMSE (% of max)**	**Deviance explained (%)**
**Taiwanese Count**	2.95 ± 0.32	46.94 ± 0.92	7.45 ± 0.74	47.64 ± 0.52
**Japanese Count**	2.38 ± 0.55	22.01 ± 0.73	3.49 ± 0.63	22.65 ± 0.54

AUC = area under the receiver operating curve (AUC). RMSE = the root mean squared error Values are mean ± standard deviation for the four model runs. Internal validation was done using ten-fold cross validation on 25% of the dataset and external validation evaluated the performance of the model on the left out 75% of the dataset.

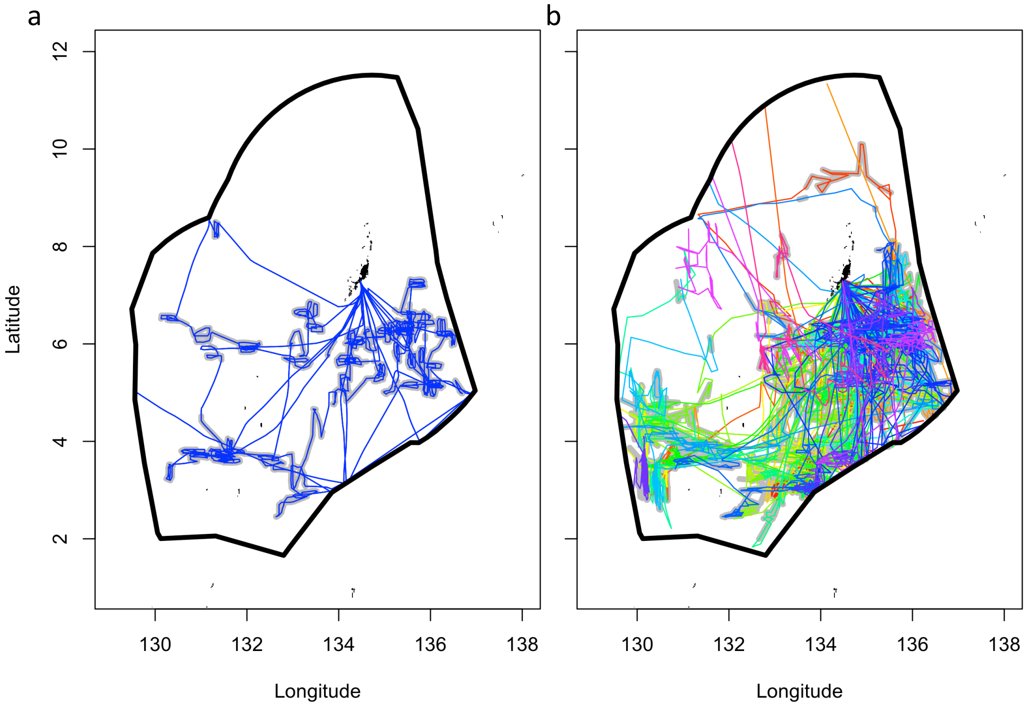

Supplemental Figure 1. An example of longline fishing vessel tracks, and transiting compared to fishing behaviors. a) An example of one vessel's track over one year and **b)** all vessel tracks over one month. Each color represents a different vessel. Gray highlights behaviors defined by our algorithm as "fishing."

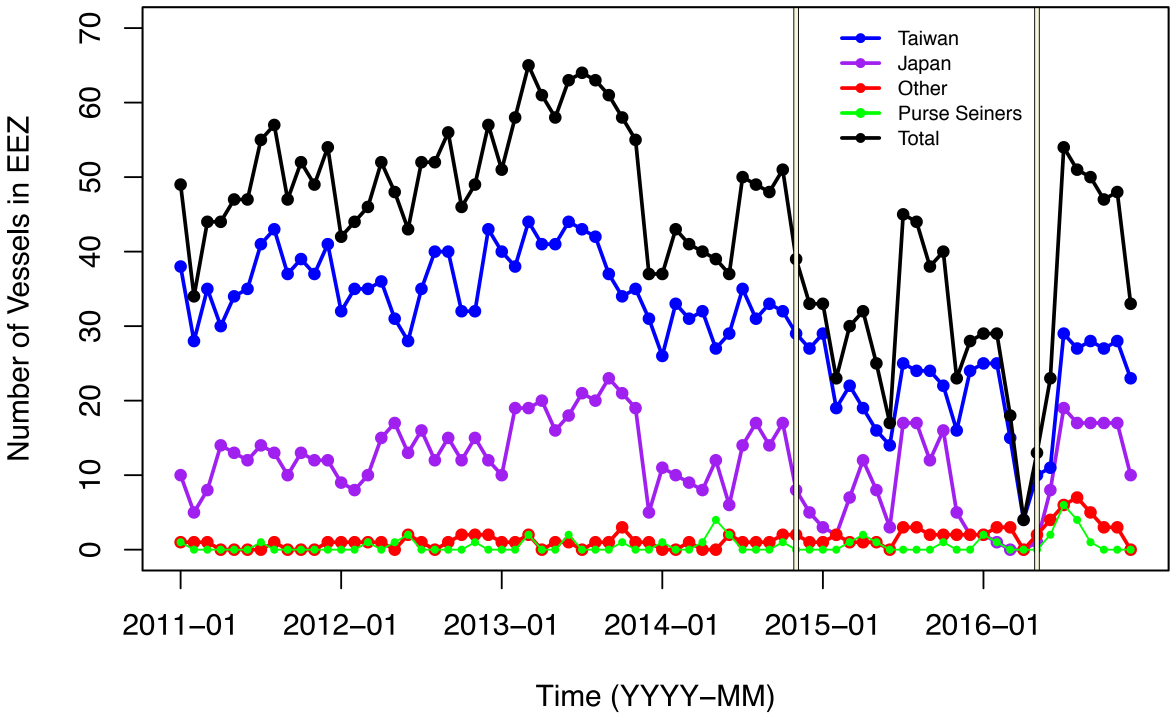

Supplemental Figure 2. The number of fishing vessels from Taiwan, Japan and other flag states in the Palau Exclusive Economic Zone (EEZ) for each month from 2011 to 2016. The total number of fishing vessels present each month is in black. The number of vessels from Japan and Taiwan were significantly correlated over time (R=0.69, $p<0.05$). The number of purse seine vessels from all countries is also shown. The yellow vertical lines indicate the El Niño period.

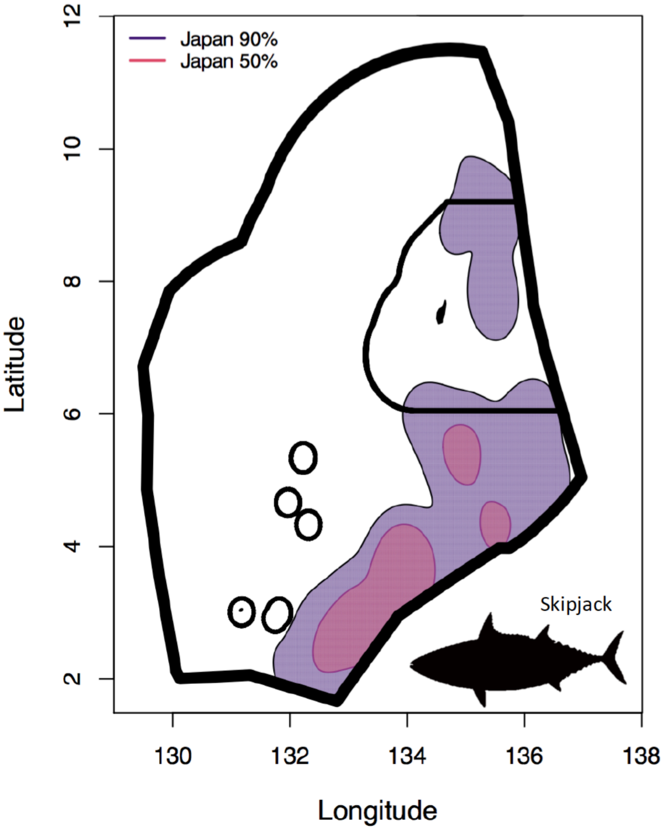

Supplemental Figure 3. Tuna fishing locations for purse seine fleet from Japan within the Palau Exclusive Economic Zone. The kernel density estimate for the 90% and 50% contour represents the range and core of fishing locations. Within the EEZ, the thin black lines represent the proposed domestic fishing zones. Skipjack tuna is the dominant species caught by the purse seine fleet.

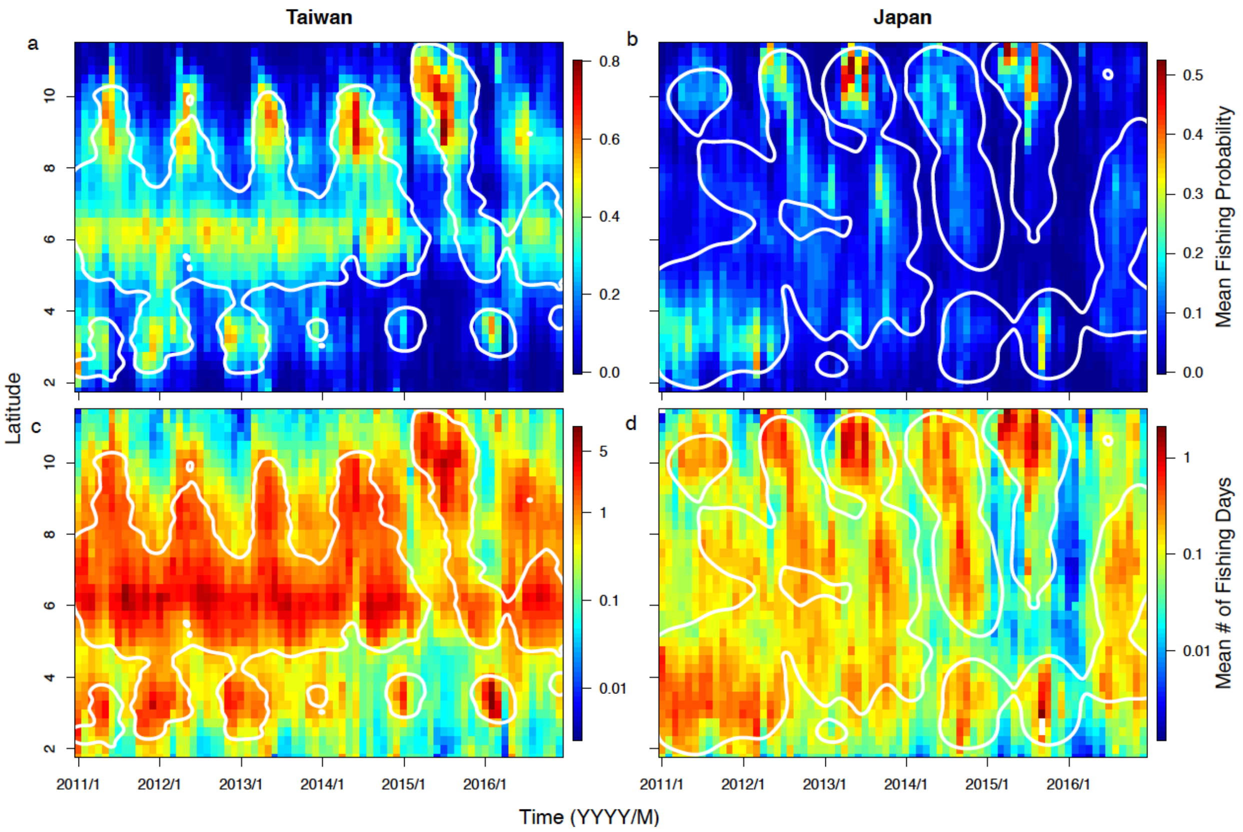

Supplemental Figure 4. Hovmöller diagrams showing boosted regression tree mean predictions. a) Mean fishing probability for Taiwanese and **b)** Japanese vessels. **c)** Mean number of fishing days for Taiwanese and **d)** Japanese vessels. The 90% kernel density estimate contour for fishing observations (from Fig. 2ab) is shown in white. The standard deviation in predictions are shown in Fig. S5.

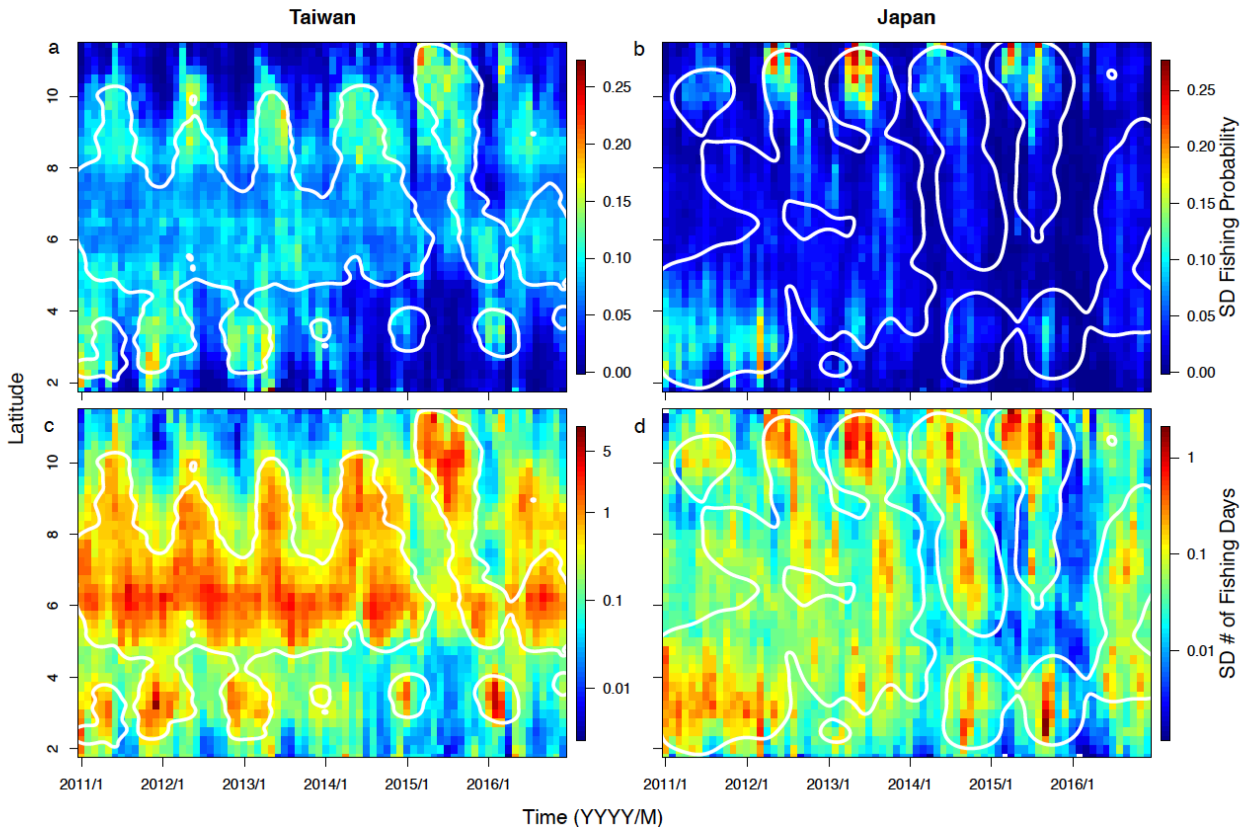

Supplemental Figure 5. Hovmöller diagrams showing the standard deviation (SD) in boosted regression tree predictions. a) SD in fishing probability for Taiwanese and **b)** Japanese vessels. **c)** SD in the number of fishing days for Taiwanese and **d)** Japanese vessels. The 90% kernel density estimate contour for fishing observations (from Fig. 2ab) is shown in white.

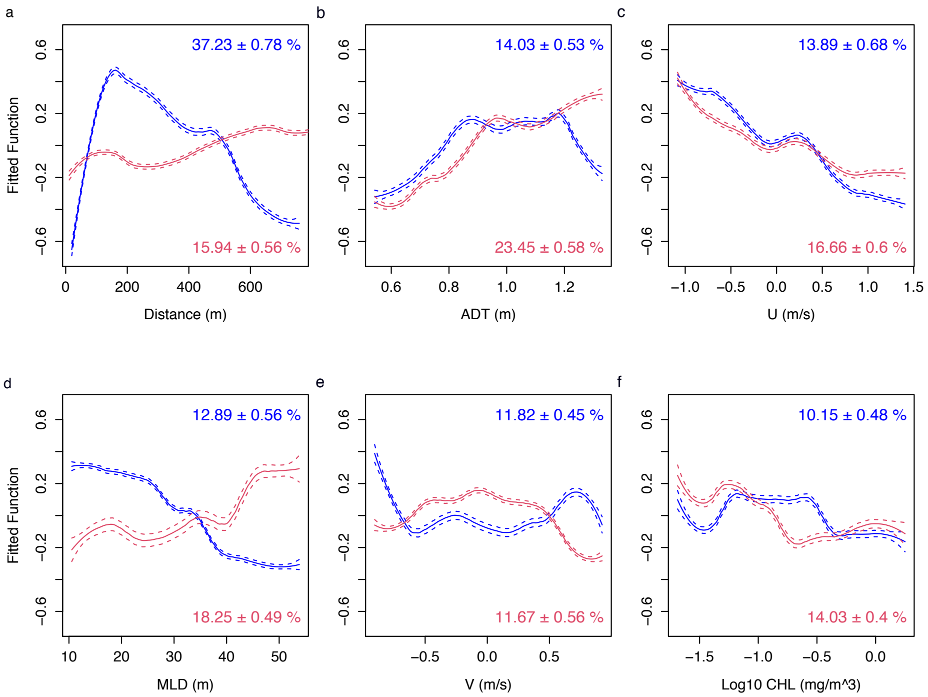

Supplemental Figure 6. Partial dependence plots of boosted regression trees (BRTs) relating Taiwanese (blue) and Japanese (pink) fishing presence/absence to six geographic or oceanographic predictors. a) Distance from Malakal Harbor for Taiwanese vessels and distance from the north EEZ entrance for Japanese vessels, **b)** absolute dynamic topography (ADT), **c)** u velocity, **d)** mixed layer depth (MLD), **e)** v velocity, and **f)** Chlorophyll A concentration (CHL). The mean (solid line) and 95% and 5% confidence interval (dashed line) are shown. Percent contribution (mean ± standard deviation) of each variable to model is shown. Partial dependence plots show the effect of a variable on the response after accounting for the average effects of other model variables.

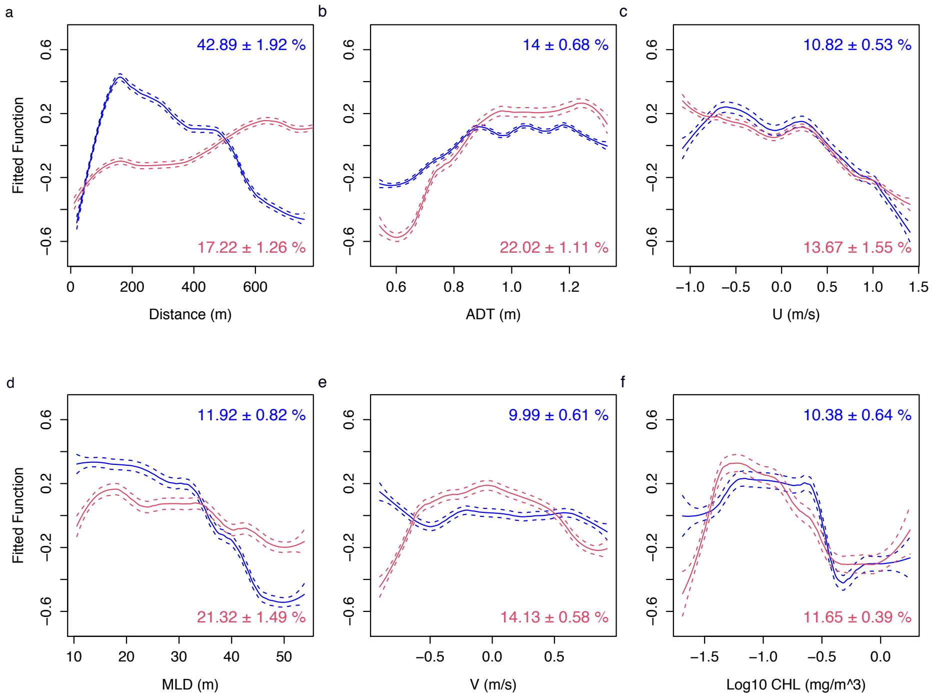

Supplemental Figure 7. Partial dependence plots of boosted regression trees (BRTs) relating Taiwanese (blue) and Japanese (pink) number of fishing days to six geographic or oceanographic predictors. a) Distance from Malakal Harbor for Taiwanese vessels and distance from the north EEZ entrance for Japanese vessels, **b)** absolute dynamic topography (ADT), **c)** u velocity, **d)** mixed layer depth (MLD), **e)** v velocity, and **f)** Chlorophyll A concentration (CHL). The mean (solid line) and 95% and 5% confidence interval (dashed line) are shown. Percent contribution (mean ± standard deviation) of each variable to model is shown. Partial dependence plots show the effect of a variable on the response after accounting for the average effects of other model variables.

Movie 1. Mean and standard deviation (SD) in Taiwanese presence/absence model predictions from January 2011 to December 2016. The points represent vessel presence per grid cell during each month.

Movie 2. Mean and standard deviation (SD) in Japanese presence/absence model predictions from January 2011 to December 2016. The points represent vessel presence per grid cell during each month.

Movie 3. Mean and standard deviation (SD) in Taiwanese count model predictions from January 2011 to December 2016. The points represent vessel presence per grid cell during each month. The number of vessels refers to the predicted cumulative number of vessels fishing that area each month.

Movie 4. Mean and standard deviation (SD) in Japanese count model predictions from January 2011 to December 2016. The points represent vessel presence per grid cell during each month. The number of vessels refers to the predicted cumulative number of vessels fishing that area each month.
